# Successful Staged Surgery for Esophagopulmonary Fistula with Lung Abscess During Neoadjuvant Chemoradiotherapy

**DOI:** 10.3390/jcm15103852

**Published:** 2026-05-16

**Authors:** Mu-Chou Lin, Wei-Lun Chang, Ying-Yuan Chen

**Affiliations:** 1 Division of Thoracic Surgery, Department of Surgery, National Cheng Kung University Hospital, College of Medicine, National Cheng Kung University, Tainan 704, Taiwan; n107963@mail.hosp.ncku.edu.tw; 2 Department of Internal Medicine, National Cheng Kung University Hospital, College of Medicine, National Cheng Kung University, Tainan 704, Taiwan; weilun1@mail.ncku.edu.tw

**Keywords:** esophagopulmonary fistula, lung abscess, esophageal cancer, neoadjuvant chemoradiotherapy, staged surgery, esophagectomy

## Abstract

Esophagopulmonary fistula (EPF) with lung abscess is a rare but serious complication during neoadjuvant chemoradiotherapy (nCRT) for esophageal squamous cell carcinoma and is often associated with poor prognosis. We report a 52-year-old man with cT3N3M0 esophageal squamous cell carcinoma who developed fever and hemoptysis during the third week of nCRT. Computed tomography showed a newly developed right lower lobe lung abscess adjacent to the primary tumor, suspicious for esophageal perforation with fistulous communication. Because the lesion and infection appeared localized, staged aggressive surgery was undertaken. The patient underwent en bloc esophagectomy and right lower lobectomy with cervical esophagostomy, followed by delayed reconstruction using a laparoscopically created gastric conduit. Final pathology showed marked treatment response with ypT1bN0 disease, and the resected lung showed no malignant involvement. R0 resection was achieved, and the patient remains disease-free at 28 months. This case illustrates a possible management pathway in a highly selected patient with localized EPF and lung abscess during nCRT, suggesting that early staged source control may preserve reconstructive options when systemic deterioration has not yet occurred.

## 1. Introduction

Neoadjuvant chemoradiotherapy (nCRT) followed by esophagectomy is the standard treatment for locally advanced esophageal squamous cell carcinoma (ESCC) [1]. This multimodal strategy improves local disease control, increases resectability, and has been associated with superior long-term survival compared with surgery alone. However, nCRT may also result in severe treatment-related complications that complicate or even preclude definitive surgical management. Among these, esophagorespiratory fistula (ERF) is one of the most devastating complications, because it may rapidly lead to pulmonary contamination, sepsis, nutritional compromise, interruption of oncologic treatment, and deterioration in performance status [1,2].

Esophagopulmonary fistula (EPF) with lung abscess represents an uncommon but particularly severe subtype of ERF. Unlike fistulas involving the central airway alone, EPF is frequently associated with infected or necrotic lung parenchyma, such that the clinical problem is not limited to fistula formation itself but also includes control of an established intraparenchymal abscess cavity that serves as a persistent reservoir of purulent and necrotic material. Once EPF develops during chemoradiotherapy, management often shifts toward palliation because the condition is commonly interpreted as a manifestation of advanced disease, treatment failure, or a physiologically unsalvageable infectious complication [3]. In this setting, esophageal stenting is often considered, but its clinical benefit may be limited when a pulmonary abscess has already formed, and definitive source control cannot be achieved by fistula occlusion alone.

Nevertheless, fistula formation during nCRT is not always a sign of uncontrolled tumor progression. In a subset of patients, it may result from treatment-related tissue damage or tumor necrosis, while resection and salvage surgery remain feasible. Distinguishing these situations is clinically important because it may determine whether treatment continues along a palliative pathway or whether salvage surgery with disease-control intent may still be considered.

We report a patient with locally advanced ESCC who developed EPF with lung abscess during nCRT and was treated with staged en bloc resection followed by delayed reconstruction. This case illustrates that, in carefully selected patients identified before systemic deterioration, early definitive surgical source control may not only achieve infection control but also preserve reconstructive options and the possibility of further cancer-directed treatment.

## 2. Case Presentation

**Figure 1:**
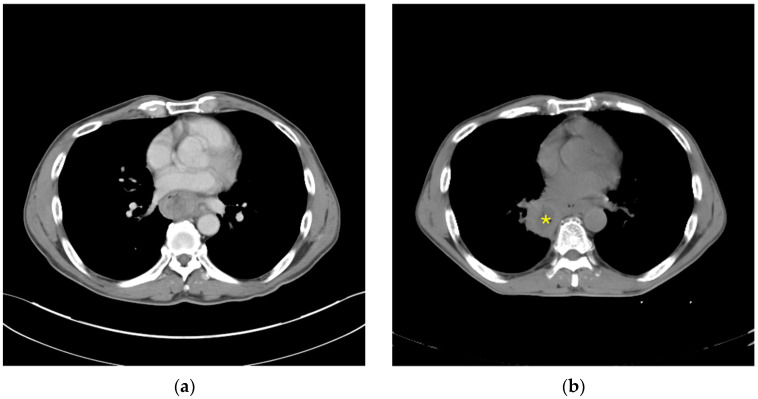
(**a**) Initial computed tomography showing an irregular enhancing tumor in the lower thoracic esophagus without definite invasion of adjacent organs.; (**b**) Computed tomography showing a right lower lobe abscess (*) adjacent to the esophageal tumor, supporting the clinical suspicion of esophageal perforation and esophagopulmonary fistula.

A 52-year-old man with a history of heavy smoking and alcohol consumption presented with progressive dysphagia and weight loss. Esophagogastroduodenoscopy revealed an ulcerative tumor in the mid-to-lower thoracic esophagus, extending from 20 to 37 cm from the incisors. Biopsy confirmed poorly differentiated squamous cell carcinoma. Contrast-enhanced chest computed tomography (CT) demonstrated an irregular enhancing esophageal mass without direct invasion of adjacent organs (Figure 1a). Extensive lymphadenopathy was identified in the subcarinal, paratracheal, and para-esophageal regions. The clinical stage was cT3N3M0. Because of severe dysphagia, a gastrostomy tube had been created before treatment to maintain enteral nutrition. The patient subsequently underwent neoadjuvant concurrent chemoradiotherapy (nCRT) with cisplatin, 5-fluorouracil, and a planned radiation dose of 50 Gy.

During the third week of nCRT, on the day scheduled for the 19th fraction of radiotherapy and before treatment delivery, the patient acutely developed high fever (up to 38.6 °C) and hemoptysis. Because of the new-onset hemoptysis, the radiation oncologist obtained an urgent radiotherapy simulation CT before that treatment session, which revealed a newly developed right lower lobe lung abscess adjacent to the esophageal tumor, with findings suspicious for esophageal perforation and fistulous communication to the right lower lobe (Figure 1b). At this time, nCRT was definitively interrupted; the patient had completed 18 fractions of radiotherapy, corresponding to a total dose of 36 Gy, and one cycle of cisplatin/5-fluorouracil. Despite these symptoms, the patient remained hemodynamically stable. Laboratory studies showed a white blood cell count of 6800/μL, lactate 1.7 mmol/L, and procalcitonin 0.07 ng/mL. Empirical broad-spectrum antimicrobial therapy with piperacillin/tazobactam (4.5 g every 6 h) was promptly initiated. Blood and sputum cultures were obtained at fever onset; blood cultures remained negative, and sputum culture yielded only normal oral flora. However, persistent spiking fever and progressive right lower lobe infiltration on serial chest radiographs indicated inadequate infection control after seven days of empirical antibiotic therapy.

**Figure 2:**
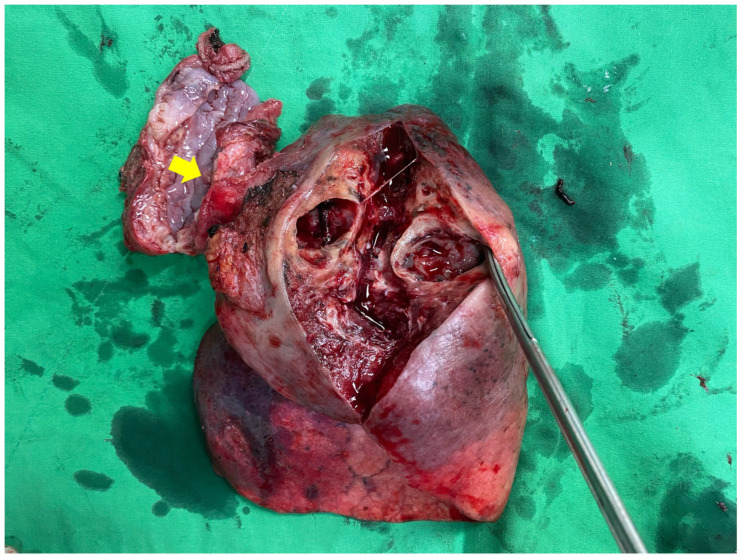
Gross specimen showing a fistulous tract (arrow) between the primary tumor and the right lower lobe, with abscess formation in the lung parenchyma.

Because imaging suggested that both the esophageal lesion and the associated lung abscess remained localized, and medical treatment failed to control the infection, a two-stage surgical strategy was adopted to achieve definitive source control while preserving the possibility of subsequent disease control. One week after symptom onset, the patient underwent right lateral thoracotomy with esophagectomy and right lower lobectomy, followed by cervical esophagostomy for diversion. Intraoperatively, severe inflammation was present between the esophageal tumor, the right lower lobe abscess, and the surrounding mediastinal tissue. The right lower lobe was densely adherent to the primary tumor at the fistula site; therefore, the adhesion was left undisturbed, and the specimen was removed en bloc to avoid contamination of the thoracic cavity. Because the priority of the first-stage procedure was infection control and diversion, extensive mediastinal lymph node dissection was not attempted under these inflammatory conditions. Gross examination of the resected specimen confirmed a direct fistulous communication between the tumor and the right lower lobe, with abscess formation in the lung parenchyma (Figure 2).

The pre-existing gastrostomy was useful for enteral nutritional support after the first-stage diversion procedure. After the initial operation, the patient received appropriate postoperative care and targeted antibiotic therapy. Intraoperative cultures obtained directly from the necrotic lung abscess cavity isolated *Parvimonas micra*, while aerobic culture showed no bacterial growth. Following this microbiological confirmation, the antimicrobial regimen was de-escalated to ampicillin/sulbactam (3 g every 6 h) postoperatively and continued for 14 days after the first-stage operation. Under this targeted therapy, the patient’s clinical infection markers improved substantially, and his fever subsided, allowing for the subsequent second-stage reconstruction. Once his fever subsided and clinical condition had stabilized, esophageal reconstruction was performed 12 days after the initial operation using a laparoscopically created gastric conduit through the substernal route. During the second-stage procedure, the gastrostomy was taken down laparoscopically without interfering with gastric conduit formation. The substernal route was selected to avoid the previously infected mediastinal and pleural field. The postoperative course after the second-stage procedure was uneventful. The patient resumed oral intake on postoperative day 7 and was discharged on postoperative day 9.

**Figure 3:**
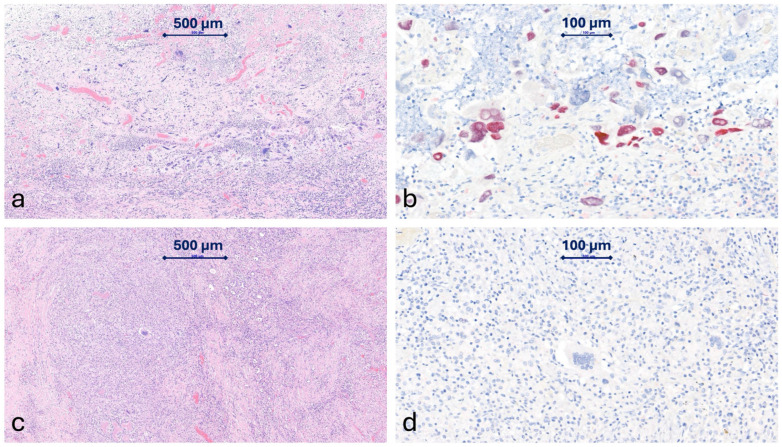
Histopathological Evaluation. (**a**) Representative sections of the esophageal mucosa show acute and chronic inflammation characterized by dense neutrophilic infiltration, focal necrosis, and foreign body giant cells (H&E stain). (**b**) Residual poorly differentiated tumor cells infiltrate the submucosal layer (ypT1b), confirmed by positive p40 immunostaining. (**c**) Sections of the right lower lobe lung show fibroblast proliferation with adhesion and necrosis. The background exhibits mild to moderate inflammatory cell infiltrate with thickened alveolar septa composed of lymphocytes, plasma cells, macrophages, and foreign body giant cells (H&E stain). (**d**) The right lower lung shows no evidence of malignancy, as demonstrated by negative p40 immunostaining.

Final pathology demonstrated a marked treatment response, with approximately 90% tumor necrosis (Figure 3). Residual poorly differentiated squamous cell carcinoma was confined to the submucosal layer, and no lymph node metastasis was identified in the right peribronchial lymph nodes examined (0/10), yielding a final pathological stage of ypT1bN0. No systematic mediastinal, abdominal, or cervical lymph node dissection was performed. The resected right lower lobe showed inflammatory change without malignant involvement, which is consistent with the hypothesis that the fistula may have resulted from rapid treatment-related tumor necrosis rather than persistent direct invasion. While this pathological response is suggestive of a treatment-related etiology, the exact underlying mechanism remains an inference based on the observed tumor regression. R0 resection was achieved.

**Figure 4:**
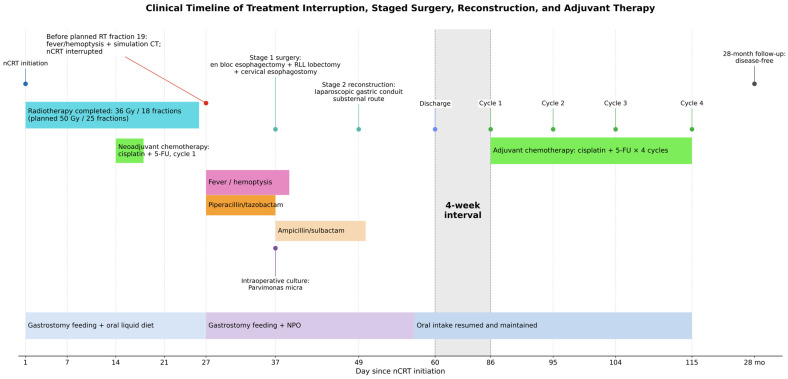
Clinical timeline of treatment interruption, antimicrobial therapy, staged surgery, reconstruction, adjuvant chemotherapy, and follow-up. The patient developed fever and hemoptysis on the day scheduled for the 19th radiotherapy fraction, before treatment delivery. Neoadjuvant chemoradiotherapy was interrupted after completion of 18 fractions, corresponding to 36 Gy, and one cycle of cisplatin/5-fluorouracil. Empirical piperacillin/tazobactam was initiated after symptom onset, followed by postoperative ampicillin/sulbactam based on intraoperative culture results. First-stage surgery consisted of esophagectomy, right lower lobectomy, and cervical esophagostomy, followed by second-stage reconstruction with a laparoscopically created gastric conduit 12 days later. The patient subsequently received four cycles of adjuvant cisplatin/5-fluorouracil and remained disease-free at 28 months of follow-up.

Following multidisciplinary team consultation, the patient received four cycles of adjuvant cisplatin and 5-fluorouracil. During 28 months of follow-up, he maintained good oral intake and nutritional status, with no evidence of tumor recurrence on surveillance imaging. The overall clinical course is summarized in Figure 4.

## 3. Discussion

Malignant ERF generally affects 5–22% of esophageal cancer patients [4]. ERF includes esophagotracheal, esophagobronchial, and esophagopulmonary fistulas (EPF). Among these, EPF is the least common subtype, accounting for only 3–11% of cases [5,6]. Because malignant ERF often occurs in the setting of advanced disease and is frequently complicated by severe infection that interrupts further oncological treatment, it has historically been managed with palliative intent [7,8,9]. Clinical management typically defaults to palliative measures, most commonly placement of self-expandable metallic stents (SEMS) [10]. However, outcomes remain poor, with reported median survival of 3.1–3.4 months for general ERFs [11,12], and only 65.5 days for EPF [3].

EPF is particularly challenging because the infectious focus often extends into the lung parenchyma, where simple fistula occlusion by stenting may be insufficient for source control. Previous reports suggest that merely occluding the fistula with an esophageal stent may sequester the infection within the lung, thereby exacerbating the septic status [3,13]. In our case, several alternative strategies were considered. Although esophageal stenting is an important option for malignant ERF, particularly in palliative settings or in patients who are not surgical candidates, it was considered less suitable in this patient because the major infectious focus was an established intraparenchymal lung abscess. Simple fistula occlusion would not directly remove or debride the necrotic abscess cavity. Similarly, percutaneous drainage might have temporarily decompressed the abscess, but it would not have addressed the fistulous communication or the necrotic esophageal tumor, raising concern for recurrent infection. Diversion alone was also considered insufficient because it would not remove the established lung abscess. Because the patient continued to have spiking fever despite seven days of empirical broad-spectrum antibiotic therapy, and because CT showed localized disease without diffuse pleural or mediastinal contamination, staged en bloc resection with diversion followed by delayed reconstruction was favored as the most direct means of source control in this highly selected setting.

The preoperative work-up of malignant EPF requires balancing anatomical detail against the risk of clinical deterioration. In this case, the diagnosis was primarily established by urgent radiotherapy simulation CT, which localized the abscess adjacent to the tumor. Additional invasive or aspiration-prone evaluations were intentionally deferred. Upper gastrointestinal endoscopy was not performed because air insufflation and gagging could worsen aspiration through the suspected fistula and aggravate pulmonary infection. Bronchoscopy was also deferred because CT suggested that the lesion and abscess were distal to the main and lobar bronchi, without evidence of central airway involvement. Contrast esophagography was not performed because of the risk of aspiration in a patient with fever and hemoptysis. In this urgent setting, CT provided sufficient anatomical information for operative planning while avoiding additional invasive or aspiration-prone investigations.

In our patient, an important element of decision making was that the exact mechanism of fistula formation was unknown at the time of surgery. Before pathological examination, we could not determine with certainty whether the fistula had resulted from progressive local disease or treatment-related necrosis. Nevertheless, this uncertainty did not diminish the need for definitive infection control. Without eliminating the source of infection, the patient would have been unable to proceed with any further oncologic treatment. Although CT-guided percutaneous drainage of the lung abscess might have provided temporary control of the abscess cavity, it would not have addressed the fistulous communication itself, and recurrent abscess formation after drain removal would have remained a substantial concern [14]. In this setting, the key practical question was not simply the mechanism of fistula formation, but whether the source of infection could still be completely removed.

Several features supported an aggressive surgical approach in this case. In our view, consideration of staged source-control surgery with curative intent in EPF depends on a structured assessment in which both physiological and anatomical prerequisites are met. In this patient, adequate physiological reserve was reflected by good baseline performance status, clear consciousness, hemodynamic stability without vasopressor or inotropic support, and absence of respiratory failure. From an anatomical perspective, CT imaging confirmed that the pulmonary sepsis was localized to a single lobe without diffuse empyema or extensive mediastinal contamination. Furthermore, because the primary tumor was initially staged as potentially resectable (cT3N3M0), we reasoned that en bloc resection could achieve definitive infection source control while preserving the possibility of subsequent disease control. By intervening before systemic deterioration or diffuse pleural contamination developed, we were able to facilitate a relatively smooth postoperative recovery after the first-stage operation.

Following the first stage, the criteria for proceeding to reconstruction included sustained resolution of spiking fever, a significant downward trend in inflammatory markers, including white blood cell count and C-reactive protein, and stabilization of chest tube drainage with transition to non-purulent serous output. The patient’s general physiological recovery, marked by independent ambulation and stable nutritional status, was also essential in determining readiness. This clinical stabilization allowed the second-stage procedure to be performed relatively early, 12 days after the first stage, thereby facilitating early nutritional restoration and minimizing potential complications associated with a prolonged cervical esophagostomy, such as fibrosis or stricture of the esophageal stump.

The final pathological findings were particularly informative. Histopathology demonstrated a marked treatment response, and the resected right lower lobe showed inflammatory change without malignant involvement. These findings were suggestive of treatment-related tumor necrosis rather than persistent direct invasion as the likely mechanism of fistula formation, although this remains an inference rather than a definitively established cause. Thus, EPF developing during nCRT should not automatically be regarded as evidence of oncologic failure or a terminal event. Although malignant fistulas often arise from direct tumor invasion into adjacent organs, particularly in cT4 disease [13,15,16], they may also result from treatment-related tissue injury and rapid tumor regression before the esophageal wall has sufficient time to remodel [2,17,18,19]. This interpretation is consistent with the report by Furuta et al., in which staged surgery for treatment-related fistulas was associated with pathological downstaging and favorable long-term survival [20].

Distinguishing between treatment-induced necrosis and local tumor progression is a critical yet challenging diagnostic task. Although pathological examination of the resected specimen remains the most reliable method for confirming the mechanism, several clinical and radiologic features may aid preoperative decision-making. In our case, the fistula developed during active nCRT, CT did not show diffuse pleural dissemination or unresectable adjacent organ invasion, and the infection remained localized to the right lower lobe. These features supported consideration of salvage surgery. Conversely, fistula formation accompanied by systemic deterioration, worsening primary tumor dimensions, new disseminated disease, or progressive invasion into unresectable adjacent structures would raise greater concern for oncologic failure. Nevertheless, without standardized markers, there remains a risk of overtreatment if aggressive surgery is applied to patients with rapidly progressive disease. Therefore, the decision for salvage surgery must be individualized, balancing the potential for disease control against the high morbidity of the procedure.

This case also highlights the value of timing. The patient remained hemodynamically stable, and intervention was undertaken before diffuse pleural contamination or systemic deterioration had developed, which likely contributed to the relatively smooth postoperative course after the first-stage operation and allowed reconstruction to be performed at an earlier stage. By contrast, if surgery is delayed until the patient has progressed to diffuse contamination or unstable sepsis, operative risk increases considerably, postoperative recovery may be prolonged, and additional damage-control procedures may become necessary, as illustrated in previous reports of surgically treated EPF with advanced infectious deterioration [13]. In this setting, delayed source control may also prolong interruption of oncologic treatment and further compromise the feasibility of reconstruction and future survival. In addition, severe pleural infection may increase concern for postoperative thoracic complications after lobectomy, including bronchial stump dehiscence or bronchopleural fistula, which, although uncommon, are potentially life-threatening [21,22].

The staged strategy also offered several practical technical advantages. Historically, surgical treatment for malignant ERF, including bypass procedures, has raised concern because of its invasiveness and the limited number of patients who are suitable candidates for such an approach [23,24]. By contrast, staged resection and diversion with delayed reconstruction simplifies the first-stage operation, shortens operative time, and allows the surgical team to focus on infection control and alimentary diversion, while reconstruction is deferred until the patient has stabilized. This strategy avoids performing an anastomosis during active inflammation or sepsis, when the risk of anastomotic leakage and other postoperative complications is increased [25,26].

In our patient, the first-stage procedure was designed primarily for infection control and alimentary diversion rather than formal oncologic dissection. Because the inflammatory reaction involving the esophageal tumor, lung abscess, and surrounding mediastinum was severe, only limited dissection was feasible, and extensive lymph node dissection was not attempted. Although R0 resection was achieved, the ypN0 status should be interpreted with caution because nodal assessment was limited to right peribronchial lymph nodes retrieved with the lobectomy specimen (0/10), rather than systematic esophageal cancer lymphadenectomy. This limited nodal assessment, together with the interrupted neoadjuvant course in which the patient received only 36 Gy of radiotherapy and one cycle of chemotherapy, was an important reason why adjuvant cisplatin and 5-fluorouracil was recommended after multidisciplinary discussion. Accordingly, the favorable long-term oncologic outcome in this patient exceeded our original expectations.

Technical planning during the first-stage procedure was also critical to the success of subsequent reconstruction. The proximal esophageal stump was intentionally preserved as long as possible to reduce tension at the future cervical anastomosis. Conversely, the distal stump was kept short while avoiding unnecessary opening of the esophageal hiatus. This strategy helped minimize subsequent inflammation and adhesions around the hiatus, thereby facilitating laparoscopic gastric mobilization during the second-stage reconstruction. Importantly, reconstruction could be achieved with a gastric conduit rather than a more complex alternative, and laparoscopic conduit creation further reduced the burden of the second-stage procedure. In our patient, pre-existing gastrostomy also facilitated enteral nutritional support after the first-stage diversion procedure. Moreover, because the gastrostomy could be taken down laparoscopically at the time of reconstruction, it did not compromise gastric conduit formation. These practical considerations underscore that early source control may influence not only survival, but also the feasibility, timing, complexity, and nutritional support strategy of subsequent reconstruction.

Taken together, this case illustrates that in highly selected patients in whom systemic deterioration has not yet occurred and en bloc resection remains technically feasible, an aggressive staged surgical approach may be a viable management pathway. While the favorable outcome in our patient suggests the potential for long-term oncologic benefit, this result should be interpreted with caution and may not be generalizable to all clinical scenarios involving EPF during neoadjuvant therapy.

## 4. Conclusions

In conclusion, although malignant EPF with a lung abscess is generally associated with poor prognosis, our case illustrates that a staged surgical strategy can be a feasible management pathway in highly selected clinical scenarios. When identified before systemic deterioration, definitive surgical source control may provide an opportunity for both infection management and subsequent reconstruction, offering a possible alternative to palliative care in cases where disease-control treatment remains a priority. However, as this report is based on a single case, these findings should not be generalized as a standard of care. Further investigation through the accumulation of cases across multiple institutions is essential to define optimal selection criteria and long-term outcomes for this aggressive surgical strategy.

## Data Availability

The original contributions presented in this study are included in the article. Further inquiries can be directed to the corresponding author.

## Abbreviations

The following abbreviations are used in this manuscript:

ERF	Esophagorespiratory fistula
nCRT	Neoadjuvant chemoradiotherapy
ESCC	Esophageal squamous cell carcinoma
EPF	Esophagopulmonary fistula
CT	Computed tomography
